# Walter Reed Army Institute of Research (WRAIR): Fifty Years of Achievements That Impact Science and Society

**DOI:** 10.1093/milmed/usaa468

**Published:** 2021-02-26

**Authors:** Carl R Alving

**Affiliations:** Laboratory of Adjuvant and Antigen Research, U.S. Military HIV Research Program, Silver Spring, MD 20910, USA

## Abstract

Thirty-four historical achievements since 1970 that emanated from scientific research at the Walter Army Institute of Research are identified and documented. Impact areas include vaccines, drug development, and clinical assays to prevent or treat infectious diseases; neuropsychiatric management of warrior performance and combat casualty; blood delivery management; and radiation protection.

## INTRODUCTION

On June 22, 2018, the WRAIR held a day-long event that celebrated “125 Years of Infectious Disease Research at WRAIR.” In the program, it was noted that “WRAIR traces its roots back to the U.S. Army Medical School, one of the first public health and preventive medicine institutions in the United States.” The program further noted that “WRAIR’s modern mission has been redefined into two research centers of excellence, the Center for Infectious Disease Research (CIDR) and the Center for Military Psychiatry and Neuroscience (CMPN).” As a further historical note that was not in the program, on July 1, 2020, I celebrated 50 years of personal service at WRAIR—a period encompassing 39% of the history of WRAIR since its founding in 1893.

Having served continuously at WRAIR since 1970, at different intervals as an active duty medical officer, or as a civilian medical officer and supervisory scientist, or as an emeritus senior scientist, I have had a unique opportunity to visualize the impact of WRAIR on science and society over a 50-year period. WRAIR is a magnificent research institution that has had a dynamic flux of military and civilian scientists working on a wide range of scientific missions to support military readiness and operations. Like any great academic research institution, WRAIR is justifiably proud of its many historic contributions that have benefited the missions of military practice and policy to support the aims of its sponsor, the U.S. Army. However, in many areas, military life is an inextricable reflection of civilian life, and some of the scientific advances coming from WRAIR as a mission-driven academic institution have also had dual-use effects on society as a whole. Here, I present a list of creative, innovative, pivotal, or enabling products; discoveries; inventions; concepts; or methods from WRAIR from 1970 through 2014 that have had an impact on military society and society in general.

### Criteria for Selection

- One or more WRAIR scientific personnel must have been clearly involved (including active duty or civilian government employees, or WRAIR-embedded contract employees). This also includes WRAIR personnel in WRAIR-associated OCONUS satellite laboratories, such as Armed Forces Research Institute of Medical Sciences in Thailand, and others.
- For all forms of *scientific* research (basic or clinical) to be included, the work must have been the first of its kind, or have opened a completely new and continuing field of scientific research, or it must have resulted in a documented unique impact on society.
- For clinical research, successful phase 3 studies or earlier phase 1 or phase 2 clinical studies that contributed to ultimate the FDA licensing of the tested product are included.
- For a new invention having importance to the public, a U.S. patent must have been issued by the U.S. Patent and Trademark Office. An issued patent serves as a regulatory announcement of commercial timing and novelty, and out-licensing demonstrates entry of the invention into an active public market. In some cases, issuance and publication of a patent is cited as evidence of the timing of a benefit to society.
- For a novel method or concept to be included, it must have been uniquely created at WRAIR and resulted in general use in society.

### Specific Exclusions

- Unsuccessful phase 3 trials, first-in-man phase 1 studies, or phase 2 studies that did not go further in the regulatory process and did not lead to regulatory approval of the tested product are excluded.
- Scientific research conducted by scientists in WRAIR or other military institutions has often resulted in outstanding purely scientific results that have been presented in the scientific literature. Although solutions to scientific problems do generally provide benefit by adding to scientific research supported by society as a whole, good science alone was not sufficient for inclusion in the present analysis unless it impacted uniquely in some way on society.

### SELECTED ACHIEVEMENTS

The accounting of 34 WRAIR achievements that resulted in beneficial results, is shown in Table I. This is admittedly a subjective exercise assembled according to the criteria described, and others might wish to add or subtract from it;however,for each item,I supply specific evidence on which it is based.

**TABLE I.** Selected Highlights in the History of WRAIR, 1970-2014^a^


1970—Development and demonstration of efficacy of the first effective meningococcal meningitis vaccine in a pivotal large-scale field trial.^1,2^
1971—Bivalent oral live adenovirus type 4 and type 7 vaccine tablets were created and FDA-approved for use in soldiers.^3–5^
The vaccine is administered to all recruits during basic training, and it retains its full protective potency for at least 6 years and probably much longer.
1975—Mefloquine (WR149240, brand name Lariam) antimalarial drug was first tested by WRAIR in humans.^6^
Mefloquine was approved by the FDA in 1989 and has been widely used for malaria prophylaxis among travelers.
1978—Synthesis of tafenoquine (WR238605), an analog of primaquine, a drug for treating and preventing relapse of *Plasmodium vivax* malaria which may occur months or years after initial infection.^7^
Tafenoquine was developed in association with SmithKline Beecham, resulting in the FDA approval in 2018.
1978—The first demonstration of the therapeutic potential of naloxone in shock and trauma. This specifically included hypovolemic shock, septic shock, spinal shock, and spinal cord injury, thus opening a new and ongoing field of scientific research.^8–13^
Naloxone (Narcan) is currently approved by the FDA as a therapeutic for opiate overdose.
1978—The first studies performed at WRAIR, establishing a new discipline in behavioral psychology called behavioral economics.^14–18^
The referenced studies are among the most cited studies in the field of behavioral economics, with applications to predict abuse liability of novel psychoactive drugs and recently to predict demand for a COVID-19 vaccine.
1978—The first discovery and description of enhanced efficacy of liposomes as drug carriers for treatment of any type of infectious disease (originally used in an animal model of visceral leishmaniasis).^19^
Liposomal amphotericin B (AmBisome; Astellas Pharma US Inc.) was approved by the FDA for treatment of visceral leishmaniasis in 1997.
U.S. patents issued to the Army inventors for above the technology in 1980 and 1981 were the 13th and 39th U.S. “liposome” patents of any type ever issued, out of 58,609 total U.S. liposome patents, as of January 20, 2020.
1984—Discovery of the genetic sequence of the circumsporozoite protein of the *Plasmodium falciparum* malaria parasite was a key historical milestone in the history of development of a successful vaccine against malaria.^20^
1985—Discovery of heterosexual transmission of HIV-1/AIDS in the United States.^21^
1986—Creation of the widely used WR staging classification for HIV-1/AIDS.^22^
1986—Creation of controlled human malaria infection (experimental infection of human volunteers) as a method for evaluating the efficacy of human malaria vaccines was a major step in the development of a successful malaria vaccine.^23^
1986—Creation of the original prototype for the composition of the inactivated hepatitis A vaccine which was later licensed for human use by the FDA in 1995.^24^
1987—Coining of the widely used term “grief leadership” in a report by the WRAIR’s Division of Neuropsychiatry about the human aftermath of the 1985 crash in Gander, Newfoundland, of a charter jet transport carrying 248 soldiers home from peacekeeping duties in the Sinai Desert.
“Leaders should provide grief leadership. Leaders who can express their sorrow, fear and sadness while continuing to function will provide a model for others to feel it acceptable to do the same.”^25–27^
1987—Identification of repeat sequence peptides (NANP/NVDP) of the circumsporozoite protein of the *Plasmodium falciparum* malaria parasite as vaccine targets.^28^ This was the key scientific breakthrough that enabled research leading to the development of the first approved human malaria vaccine (RTS,S; Mosquirix, GSK).
1987—Performed the first-ever human injection and evaluation of a malaria vaccine containing a recombinant protein.^29^
A later improved vaccine containing a related recombinant protein (RTS,S; Mosquirix, GSK) was approved for large-scale testing under European Medicines Agency and World Health Organization auspices for prevention of malaria in infants in 3 sub-Saharan countries in Africa.
COL Franklin K. Top, MC, USA (WRAIR director and commandant) was the first person injected.
1988—Halofantrine (WR171669, brand name Halfan), a blood antimalarial schizontozide drug effective against chloroquine- and sulfonamide/pyrimethamine-resistant malaria was first tested in humans in 1988.^30^ Halofantrine was licensed for human use by the FDA in 1992.
1989—Performed the first-ever human injection and evaluation of a liposome-based candidate vaccine against an infectious disease (1989-1992).^31^
This also was the first-ever human injection of liposomes containing monophosphoryl lipid A and the first-ever human injection of a liposome-based malaria vaccine. COL Carl R. Alving, MC, USA was the first person injected.
1991—Creation of the WR repository and survey of compounds from the antimalarial drug development program.^32^
The WR drug collection, overseen by the WRAIR Division of Experimental Therapeutics, comprises >800,000 highly characterized compounds that are physically maintained in a repository in Rockville, MD. Each drug is identified by a unique WR number.
1994—WRAIR (AFRIMS) served as the lead agency in the collaborative pivotal phase 3 trial for hepatitis A vaccine leading to licensing for human use by the FDA in 1995.^33^
1995—Amifostine (WR2721) (Ethyol), the first broad-spectrum cytoprotectant approved in many countries for clinical use, is an analog of cysteamine and was originally developed by WRAIR in the 1950s as a radioprotective agent. It was approved for human use by the FDA in 1995 for protection of normal tissue during cancer radiation treatment.^34,35^
1998—Discovery and founding of the field of transcutaneous immunization by skin patch; the invention, patenting, development, and out-licensing of the technology for vaccine application to Iomai Corp.^36–43^
1999—Invention, patenting, demonstration of efficacy in 2003, and commercialization in 2011, of the Lethal Mosquito Breeding Container, known as Trap-N-Kill.^44–47^
Product was distributed for reduction of *Aedes aegypti* mosquito populations.
1999—Invention, internal development, and demonstration of efficacy (in phase 3 testing) (2013) of WR279396, consisting of an aminoglycoside cream containing 15% paromomycin sulfate in a complex base to aid drug penetration for treatment of cutaneous leishmaniasis.^48–51^
2000—Invention of the basis of the SOLX System for prolonged blood storage (LEUKOSEP HWB-600-XL Leukocyte Reduction Filtration System for Whole Blood with CPD Anticoagulant and SOLX).^52,53^ The system was approved for human use by the FDA in 2013.
2000—Atovaquone/proguanil (Malarone) was approved by the FDA for treatment or prevention of malaria in 2002. Development of drug combination strategies, dose-ranging preclinical and clinical studies, and pivotal efficacy trials were organized by WRAIR in partnership with GlaxoSmithKline.^54^
2001—Invention of system and method for predicting human cognitive performance using data from an actigraph in 2001 that led to 2B‐Alert App: A mobile application for real‐time individualized prediction of alertness in 2019.^55–57^
2003—Invention of a system and method to predict human performance based on sleep history and circadian rhythm, called the SAFTE model.^58–61^
The SAFTE model was subsequently commercialized under license from the Army and is currently used by all the major U.S. Airlines and over 60 aviation and other organizations worldwide for predicting and managing fatigue in transportation and shiftwork. It has been incorporated into transportation policy by the Federal Railroad Administration and the Federal Aviation Administration.
2005—WRAIR conducted the pivotal large-scale field trial that validated the BinaxNow malaria rapid diagnostic test that was approved by the FDA in 2007.^62–64^
2009—WRAIR served as the lead agency in the collaborative first phase 2 efficacy testing of RTS,S/AS01 vaccine that subsequently became the first licensed malaria vaccine (Mosquirix, GSK).^65^
2009—WRAIR (AFRIMS) was the lead agency, together with many others, in the joint RV144 phase 3 trial of the first-ever, and only-to-date, HIV-1 vaccine to demonstrate protection against HIV infection. The trial involved more than 16,000 volunteers in Thailand, half vaccinated and half not vaccinated as a control group.^66^
RV144 was rated number 2 on TIME magazine’s Top 10 Medical Breakthroughs list for 2009 and number 8 on their 50 Best Inventions of the Year.
“RV144 was the light at the time in the field, without which we may have given up,” said Dr. John Mascola, director of the National Institutes of Health Vaccine Research Center on World AIDS Day at WRAIR, on November 26, 2019. “In the last 10 years of HIV vaccine progress, RV144 is the anchor.”
2009—Created Vero cell-derived inactivated Japanese encephalitis virus technology that led to licensing of JEV vaccine (IXIARO, Intercell) for human use by the FDA in 2009.^67^
2013—Created, developed, patented, and out-licensed a novel immunotherapeutic vaccine technology against heroin and other opiates in collaboration with the National Institute on Drug Abuse and the National Institute on Alcohol Abuse and Alcoholism.^68–70^
2014—WRAIR investigators were the first to test in humans the subsequently FDA-licensed Ebola vaccine, rVSV∆G-ZEBOV-GP (Ervebo, Merck).^71–73^
2014—The CL Detect Rapid Test for Cutaneous Leishmaniasis. The CL Detect was developed by the Army in partnership with InBios International, Inc., the Institut Pasteur de Tunis in Tunisia, WRAIR, and the U.S. Army Small Business Innovation Research Program, and was approved by the FDA in 2014.^74^

a The listing of achievements in this table was prepared in 2020 but was limited to 2014 for technical reasons of historical delay. Hundreds of more recent and ongoing research projects at WRAIR, many of which include multi-year clinical research trials, new inventions and innovations under development, and basic research, are expected to emerge to add to the historical record, with the time of recognized future impact beyond 2020.

Abbreviations: AFRIMS, Armed Forces Research Institute of Medical Sciences; SAFTE, Sleep, Activity, Fatigue, and Task Effectiveness; WR, Walter Reed.

## DISCUSSION

WRAIR is a large post with a complex organization and broad mission. The authorized strength in CY 1990 was 798 military and civilian employees, many of whom were physicians and scientists, and this did not include numerous contractors. Hundreds of scientific publications and technical reports are generated each year, but among the vastness of output, only a few items are ultimately recognized over a period of time as having an impact on science or society. Some achievements are recognized immediately: The successful phase 3 RV144 Thai HIV vaccine trial quickly changed the direction of the entire HIV vaccine field and gave hope to the public that an HIV vaccine was possible. Other achievements required years or even decades to achieve recognition: The 1995 FDA approval of amifostine (WR2721) as a drug for protecting normal tissue during radiation treatment of cancer came four decades after synthesis of the anti-radiation compound at WRAIR in the 1950s. Because of the inherent delay that often occurs in the process of historical recognition, the 50-year perspective presented in Table I pauses in 2014 as we await the emergence of historical impacts of the many recent and ongoing research efforts now being undertaken.

Failure to achieve an impact on science or society can have many different causes. The overall statistical “probability of success” for regulatory approval of candidate vaccines to infectious diseases overall is estimated to be only 33.4%.^75^ Therefore, with reluctance, I have excluded many first-in-human phase 1 trials of interesting and exciting candidate vaccines, including those to chikungunya virus,^76^ Ebola and Marburg viruses,^77^ Middle East respiratory syndrome virus,^78,79^ and Zika virus,^80^ because they have not yet been proved successful in a phase 3 trial and have not been licensed for human use by a national regulatory agency.

Scientific research is a competitive exercise, often requiring collaboration with groups of scientists at different institutions or companies. For example, the licensing by the FDA of the first Ebola virus vaccine resulted from competition among multiple different collaborative groups of scientists (including from WRAIR),^71^ with the winner of the competition (Merck) taking the prize that benefits all.^81^ The frenzied international recent attempts by more than 130 commercial and research groups to develop an effective SARS-CoV-2 vaccine provides a further example of such competition, and WRAIR is deeply immersed with its own military COVID-19 vaccine candidate.^82^ WRAIR is also at the forefront of discussions about the possible use, and the ethics, of controlled human infection for accelerating SARS-CoV-2 vaccine development.^83^ This is a model that is ethically informed from a historical standpoint, in part, by the controlled human malaria infection model developed at WRAIR in 1986 that has been highly useful and beneficial for malaria vaccine development.^23^

WRAIR is competitive at the highest level as a scientific enterprise, and because of its mission needs, unique technology, and OCONUS capabilities, it is sometimes a linchpin research partner for investigating health problems that distinctively afflict both military and civilian personnel.

## References

[1] Artenstein MS , Gold R, Zimmerly JG, et al: Prevention of meningococcal disease by group C polysaccharide vaccine. N Engl J Med 1970; 282(8): 417-20. doi: 10.1056/NEJM197002192820803 4983754

[2] Artenstein AW , Opal JM, Opal SM, et al: History of U.S. military contributions to the study of vaccines against infectious diseases. Mil Med 2005; 170(4Suppl): 3-11. doi: 10.7205/milmed.170.4s.3 15916278

[3] Top FH Jr , Grossman RA, Bartelloni PJ, et al: Immunization with live types 7 and 4 adenovirus vaccines. I. Safety, infectivity, antigenicity, and potency of adenovirus type 7 vaccine in humans. J Infect Dis 1971; 124(2): 148-54. 4330997 10.1093/infdis/124.2.148

[4] Top FH Jr , Buescher EL, Bancroft WH, Russell PK: Immunization with live types 7 and 4 adenovirus vaccines. II. Antibody response and protective effect against acute respiratory disease due to adenovirus type 7. J Infect Dis 1971; 124(2): 155-60. 4330998 10.1093/infdis/124.2.155

[5] Collins ND , Adhikari A, Yang Y, et al: Live oral adenovirus type 4 and type 7 vaccine induces durable antibody response. Vaccines (Basel) 2020; 8(3): E411. doi: 10.3390/vaccines8030411. PMC7564809 32718082

[6] Trenholme CM , Williams RL, Desjardins RE, et al: Mefloquine (WR 142,490) in the treatment of human malaria. Science 1975; 190(4216): 792-4. doi: 10.1126/science.1105787 1105787

[7] Milhous W : Development of new drugs for chemoprophylaxis of malaria. Bull Soc Pathol Exot 2001; 94(2 Pt 2): 149-51. 16579068

[8] Holaday JW , Faden AI: Naloxone reversal of endotoxin hypotension suggests role of endorphins in shock. Nature 1978; 275(5679): 450-1. doi: 10.1038/275450a0. 357994

[9] Faden AI , Holaday JW: Opiate antagonists: a role in the treatment of hypovolemic shock. Science 1979; 205(4403): 317-8. doi: 10.1126/science.451606. 451606

[10] Faden AI , Jacobs TP, Holaday JW: Naloxone alteration of physiological parameters in spinally transected animals. Trans Am Neurol Assoc 1979; 104: 157-61. 553396

[11] Holaday JW , Faden AI: Naloxone acts at central opiate receptors to reverse hypotension, hypothermia and hypoventilation in spinal shock. Brain Res 1980; 189(1): 295-300. doi: 10.1016/0006-8993(80)90032-3 6244878

[12] Reynolds DG , Gurll NJ, Vargish T, Lechner RB, Faden AI, Holaday JW: Blockade of opiate receptors with naloxone improves survival and cardiac performance in canine endotoxic shock. Circ Shock 1980; 7(1): 39-48. 6248265

[13] Holaday JW , Faden AI: Naloxone treatment in shock. Lancet 1981; 2(8239): 201. doi: 10.1016/s0140-6736(81)90381-0. 6114265

[14] Hursh SR : The economics of daily consumption controlling food- and water-reinforced responding. J Exp Anal Behav 1978; 29(3): 475-91. doi: 10.1901/jeab.1978.29-475. 16812071 PMC1332845

[15] Hursh SR : Economic concepts for the analysis of behavior. J Exp Anal Behav 1980; 34(2): 219-38. doi: 10.1901/jeab.1980.34-219. 16812188 PMC1332999

[16] Hursh SR , Natelson BH: Electrical brain stimulation and food reinforcement dissociated by demand elasticity. Physiol Behav 1981; 26(3): 509-15. doi: 10.1016/0031-9384(81)90180-3. 7243966

[17] Hursh SR : Behavioral economics. J Exp Anal Behav 1984; 42(3): 435-52. doi: 10.1901/jeab.1984.42-435. 16812401 PMC1348114

[18] Hursh SR : Behavioral economics of drug self-administration and drug abuse policy. J Exp Anal Behav 1991; 56(2): 377-93. doi: 10.1901/jeab.1991.56-377. 1955823 PMC1323109

[19] Alving CR , Steck EA, Chapman WL Jr, et al: Therapy of leishmaniasis: superior efficacies of liposome-encapsulated drugs. Proc Natl Acad Sci U S A 1978; 75(6): 2959-63. 208079 10.1073/pnas.75.6.2959 PMC392686

[20] Dame JB , Williams JL, McCutchan TF, et al: Structure of the gene encoding the immunodominant surface antigen on the sporozoite of the human malaria parasite *Plasmodium falciparum*. Science 1984; 225(4662): 593-9. 6204383 10.1126/science.6204383

[21] Redfield RR , Markham PD, Salahuddin SZ, et al: Frequent transmission of HTLV-III among spouses of patients with AIDS-related complex and AIDS. JAMA 1985; 253(11): 1571-3. 2983127

[22] Redfield RR , Wright DC, Tramont EC: The Walter Reed staging classification for HTLV-III/LAV infection. N Engl J Med 1986; 314(2): 131-2. 2934633 10.1056/NEJM198601093140232

[23] Chulay JD , Schneider I, Cosgriff TM, et al: Malaria transmitted to humans by mosquitoes infected from cultured *Plasmodium falciparum*. Am J Trop Med Hyg 1986; 35(1): 66-8. 3511753 10.4269/ajtmh.1986.35.66

[24] Binn LN , Bancroft WH, Lemon SM, et al: Preparation of a prototype inactivated hepatitis A virus vaccine from infected cell cultures. J Infect Dis 1986; 153(4): 749-56. 3005435 10.1093/infdis/153.4.749

[25] Bartone P , Cervantes Jr RA, Garrigan JJ, et al: The human response to the Gander military air disaster: a summary report. (Report WRAIR-NP-88-12), Department of Military Psychiatry, Division of Neuropsychiatry, Walter Reed Army Institute of Research, U.S. Army Medical Research and Development Command, Ft. Detrick, MD, 12 1987.

[26] Smith AL : Coping with death and grief: a strategy for army leadership. Strategy Research Project, US Army War College, Carlisle Barracks, Pennsylvania, 7 April 1999. Available at https://apps.dtic.mil/dtic/tr/fulltext/u2/a361181.pdf; accessed September 24, 2020.

[27] Chang E : With the world in crisis, consider practicing ‘grief leadership.’ Available at https://www.washingtonpost.com/lifestyle/wellness/covid-grief-leadership/2020/09/16/51210be4-f444-11ea-bc45-e5d48ab44b9f_story.html; accessed September 24, 2020.

[28] Dame JB , Williams JL, McCutchan TF, Schneider I: Immunologically active peptides capable of inducing immunization against malaria and genes encoding therefor. U.S. Patent No. 4,707,357, issued November 17, 1987.

[29] Ballou WR , Hoffman SL, Sherwood JA, et al: Safety and efficacy of a recombinant DNA *Plasmodium falciparum* sporozoite vaccine. Lancet 1987; 1(8545): 1277-81. 2884410 10.1016/s0140-6736(87)90540-x

[30] Boudreau EF , Pang LW, Dixon KE, et al: Malaria: treatment efficacy of halofantrine (WR 171,669) in initial field trials in Thailand. Bull World Health Organ 1988; 66(2): 227-35. 3293828 PMC2491042

[31] Fries LF , Gordon DM, Richards RL, et al: Liposomal malaria vaccine in humans: a safe and potent adjuvant strategy. Proc Natl Acad Sci U S A 1992; 89(1): 358-62. 1729706 10.1073/pnas.89.1.358 PMC48236

[32] Sweeney TR : A survey of compounds from the antimalarial drug development program of the U.S. Army Medical Research and Development Command. Published in two volumes by WRAIR in April 1991.

[33] Innis BL , Snitbhan R, Kunasol P, et al: Protection against hepatitis A by an inactivated vaccine. JAMA 1994; 271(17): 1328-34. 8158817

[34] FDA 1995 approval of amifostine. Available at https://www.accessdata.fda.gov/scripts/cder/daf/index.cfm?event=overview.process&ApplNo=020221; accessed on September 23, 2020.

[35] Capizzi RL , Oster W: Chemoprotective and radioprotective effects of amifostine: an update of clinical trials. Int J Hematol 2000; 72(4): 425-35. 11197208

[36] Glenn GM , Rao M, Matyas GR, Alving CR: Skin immunization made possible by cholera toxin. Nature 1998; 391(6670): 851. 10.1038/36014 9495336

[37] Glenn GM , Scharton-Kersten T, Vassell R, et al: Transcutaneous immunization with cholera toxin protects mice against lethal mucosal toxin challenge. J Immunol 1998; 161(7): 3211-4. 9759833

[38] Alving CR , Glenn GM: Transdermal delivery system for antigen, U.S. Patent No. 5,910,306, issued June 8, 1999.

[39] Glenn GM , Alving CR: Adjuvant for transcutaneous immunization. U.S. Patent No. 5,980,898, issued November 9, 1999.

[40] Glenn GM , Alving CR: Use of penetration enhancers and barrier disruption agents to enhance the transcutaneous immune response. U.S. Patent No. 6,797,276, issued 28 September 2004.

[41] Glenn GM , Alving CR: Adjuvant for transcutaneous immunization. U.S. Patent No. 7,037,499, issued 2 May 2006.

[42] Glenn GM , Alving CR: Use of penetration enhancers and barrier disruption methods to enhance the immune response of antigen and adjuvant. U.S. Patent No. 7,378,097, issued 27 May 2008.

[43] Glenn GM , Alving CR: Transcutaneous immunization without heterologous adjuvant. U.S. Patent No. 8,911,742, issued 16 December 2014.

[44] Perich MJ , Zeichner BC: Lethal mosquito breeding container, U.S. Patent No. 5,983,557, issued Nov. 16, 1999.

[45] Perich MJ , Kardec A, Braga IA, et al: Field evaluation of a lethal ovitrap against dengue vectors in Brazil. Med Vet Entomol 2003; 17(2): 205-10. 12823838 10.1046/j.1365-2915.2003.00427.x

[46] Sithiprasasna R , Mahapibul P, Noigamol C, et al: Field evaluation of a lethal ovitrap for the control of *Aedes aegypti* (Diptera: culicidae) in Thailand. J Med Entomol 2003; 40(4): 455-62. 14680111 10.1603/0022-2585-40.4.455

[47] Baragona S : Army laboratories work together to defeat dengue. Army media publication. 2011. Available at https://www.army.mil/article/51064/army_laboratories_work_together_to_defeat_dengue; accessed September 21, 2020.

[48] Grogl M , Schuster BG, Ellis WY, Berman JD: Successful topical treatment of murine cutaneous leishmaniasis with a combination of paromomycin (aminosidine) and gentamicin. J Parasitol 1999; 85(2): 354-9. 10219319

[49] Grogl M , Fleckenstein L, McGreevy P, Schuster B: Antileishmanial composition for topical application. U.S. Patent No. 6,284,739, issued September 4, 2001.

[50] Ben Salah A , Ben Messaoud N, Guedri E, et al: Topical paromomycin with or without gentamicin for cutaneous leishmaniasis. N Engl J Med 2013; 368(6): 524-32. 23388004 10.1056/NEJMoa1202657

[51] Ben Salah A , Zaâtour A, Ben Messaoud N, et al: Parasite load decrease during application of a safe and easily applied antileishmanial aminoglycoside cream. PLoS Negl Trop Dis 2014; 8(5): e2749. 10.1371/journal.pntd.0002749 PMC4031053 24853096

[52] Hess JR , Greenwalt TJ: Prolonged storage of red blood cells and composition, US Patent No. 6,150,085, issued Nov 21, 2000.

[53] USAMMDA public affairs. FDA approves new drug application for whole blood collection system. July 1, 2013. Available at https://www.army.mil/article/105753/FDA_Approves_New_Drug_Application_for_Whole_Blood_Collection_System/; accessed September 23, 2020.

[54] Kitchen LW , Vaughn DW, Skillman DR: Role of US military research programs in the development of US Food and Drug Administration–approved antimalarial drugs. Clin Infect Dis 2006; 43(1): 67-71. 16758420 10.1086/504873

[55] Balkin TJ , Belenky GL, Hall SW, et al: System and method for predicting human cognitive performance using data from an actigraph. U.S. Patent No. 6,241,686, issued June 5, 2001

[56] Reifman J , Kumar K, Wesensten NJ, et al: 2B-Alert Web: an open-access tool for predicting the effects of sleep/wake schedules and caffeine consumption on neurobehavioral performance. Sleep 2016; 39(12): 2157-9. 27634801 10.5665/sleep.6318 PMC5103804

[57] Reifman J , Ramakrishnan S, Liu J, et al: 2B-Alert App: a mobile application for real-time individualized prediction of alertness. J Sleep Res 2019; 28(2): e12725. doi: 10.1111/jsr.12725. PMC7378949 30033688

[58] Hursh SR : System and method for evaluating task effectiveness based on sleep pattern. U.S. Patent No. 6,579,233, issued 17 June 2003. Invention was made during a collaborative contract with WRAIR.

[59] Hursh SR , Redmond DP, Johnson ML, et al: Fatigue models for applied research in warfighting. Aviat Space Environ Med 2004; 75(3, Sect II): 44-53. 15018265

[60] Hursh SR , Balkin TJ, Miller JC, Eddy DR: The fatigue avoidance scheduling tool: modeling to minimize the effects of fatigue on cognitive performance. SAE Transactions 2004; 113(1): 111-19. [SAE, Society of Automobile Engineers]

[61] Hursh SR , Elsmore T, Eddy DR: Interface for a system and method for evaluating task effectiveness based on sleep pattern. U.S. Patent No. 7,384,394, issued 10 June 2008.

[62] Murray CK , Bell D, Gasser RA, Wongsrichanalai C: Rapid diagnostic testing for malaria. Trop Med Int Health 2003; 8(10): 876-83. 14516298 10.1046/j.1365-3156.2003.01115.x

[63] Gasser RA Jr , Magill AJ, Ruebush T, et al: 2005. Malaria diagnosis: performance of NOW ICT Malaria in a large scale field trial. Abstr. 54th Annu. Meet. Am. Soc. Trop. Med. Hyg., abstr. 2338.

[64] Murray CK , Gasser RA Jr, Magill AJ, Miller RS: Update on rapid diagnostic testing for malaria. Clin Microbiol Rev 2008; 21(1): 97-110. 18202438 10.1128/CMR.00035-07 PMC2223842

[65] Kester KE , Cummings JF, Ofori-Anyinam O, et al: RTS,S Vaccine Evaluation Group. Randomized, double-blind, phase 2a trial of falciparum malaria vaccines RTS,S/AS01B and RTS,S/AS02A in malaria-naive adults: safety, efficacy, and immunologic associates of protection. J Infect Dis 2009; 200(3): 337-46. 19569965 10.1086/600120

[66] Rerks-Ngarm S , Pitisuttithum P, Nitayaphan S, et al: Vaccination with ALVAC and AIDSVAX to prevent HIV-1 infection in Thailand. N Engl J Med 2009; 361(23): 2209-20. 19843557 10.1056/NEJMoa0908492

[67] Jelinek T : IXIARO updated: overview of clinical trials and developments with the inactivated vaccine against Japanese encephalitis. Expert Rev Vaccines 2013; 12(8): 859-69. 23984958 10.1586/14760584.2013.835638

[68] Matyas GR , Mayorov AV, Rice KC, et al: Liposomes containing monophosphoryl lipid A: a potent adjuvant system for inducing antibodies to heroin hapten analogs. Vaccine 2013; 31(26): 2804-10. 23624097 10.1016/j.vaccine.2013.04.027 PMC4120113

[69] Alving CR , Matyas GR, Mayorov AV, et al: Induction of highly specific antibodies to a hapten but not to a carrier peptide by immunization, U.S. Patent No. 9,193,739, issued 24 November 2015.

[70] Opiant. OPNT005 Heroin Vaccine: hapten + liposome adjuvant. Available at https://www.opiant.com/products-pipeline/pipeline/opnt005-heroin-vaccine-hapten-liposome-adjuvant/; accessed September 23, 2020.

[71] Heppner DG Jr , Kemp TL, Martin BK, et al: Safety and immunogenicity of the rVSV∆G-ZEBOV-GP Ebola virus vaccine candidate in healthy adults: a phase 1b randomised, multicentre, double-blind, placebo-controlled, dose-response study. Lancet Infect Dis 2017; 17(8): 854-66. doi: 10.1016/S1473-3099(17)30313-4. 28606591

[72] Monath TP , Fast PE, Modjarrad K, et al: rVSVΔG-ZEBOV-GP (also designated V920) recombinant vesicular stomatitis virus pseudotyped with Ebola Zaire Glycoprotein: standardized template with key considerations for a risk/benefit assessment. Vaccine X 2019 Jan 29; 1: 100009. doi: 10.1016/j.jvacx.2019.100009. PMC6668225 31384731

[73] Branswell H : ‘Against all odds’: the inside story of how scientists across three continents produced an Ebola vaccine. Available at https://www.statnews.com/2020/01/07/inside-story-scientists-produced-world-first-ebola-vaccine/; accessed September 22, 2020.

[74] Phillips C : Leishmania Rapid Diagnostic Device Receives FDA Clearance. Available at https://www.army.mil/article/138447/leishmania_rapid_diagnostic_device_receives_fda_clearance; accessed October 15, 2020.

[75] Wong CH , Siah KW, Lo AW: Estimation of clinical trial success rates and related parameters. Biostatistics 2019; 20(2): 273-86. doi: 10.1093/biostatistics/kxx069. Erratum in: Biostatistics. 2019 Apr 1;20(2):366 29394327 PMC6409418

[76] Harrison VR , Eckels KH, Bartelloni PJ, Hampton C: Production and evaluation of a formalin-killed chikungunya vaccine. J Immunol 1971; 107(3): 643-7. 4999088

[77] Kibuuka H , Berkowitz NM, Millard M, et al: Safety and immunogenicity of Ebola virus and Marburg virus glycoprotein DNA vaccines assessed separately and concomitantly in healthy Ugandan adults: a phase 1b, randomised, double-blind, placebo-controlled clinical trial. Lancet 2015; 385(9977): 1545-54. doi: 10.1016/S0140-6736(14)62385-0. 25540891

[78] Pellerin C : Army Scientists Begin First MERS Vaccine Clinical Trial. DOD NEWS 2016. Available at https://www.defense.gov/Explore/News/Article/Article/657176/army-scientists-begin-first-mers-vaccine-/; accessed September 21, 2020.

[79] Modjarrad K , Roberts CC, Mills KT, et al: Safety and immunogenicity of an anti-Middle East respiratory syndrome coronavirus DNA vaccine: a phase 1, open-label, single-arm, dose-escalation trial. Lancet Infect Dis 2019; 19(9): 1013-22. doi: 10.1016/S1473-3099(19)30266-X. 31351922 PMC7185789

[80] Stephenson KE , Tan CS, Walsh SR, et al: Safety and immunogenicity of a Zika purified inactivated virus vaccine given via standard, accelerated, or shortened schedules: a single-centre, double-blind, sequential-group, randomised, placebo-controlled, phase 1 trial. Lancet Infect Dis 2020; 20(9): 1061-70. doi: 10.1016/S1473-3099(20)30085-2. 32618279 PMC7472641

[81] Wolf J , Bruno S, Eichberg M, et al: Applying lessons from the Ebola vaccine experience for SARS-CoV-2 and other epidemic pathogens. NPJ Vaccines 2020 Jun 15; 5: 51. doi: 10.1038/s41541-020-0204-7. PMC7295741 32566261

[82] U.S. Army Advances Lead COVID-19 Vaccine Candidate. Available at https://www.wrair.army.mil/node/499; accessed September 29, 2020.

[83] Deming ME , Michael NL, Robb M, Cohen MS, Neuzil KM: Accelerating development of SARS-CoV-2 vaccines—the role for controlled human infection models. N Engl J Med 2020; 383(10): e63. doi: 10.1056/NEJMp2020076. Epub 2020 Jul 1. PMC7968616 32610006

